# Large-Scale Encapsulation of Magnetic Iron Oxide Nanoparticles via Syngas Photo-Initiated Chemical Vapor Deposition

**DOI:** 10.1038/s41598-018-30802-1

**Published:** 2018-08-15

**Authors:** Donya Farhanian, Gregory De Crescenzo, Jason R. Tavares

**Affiliations:** CREPEC, Department of Chemical Engineering, École Polytechnique de Montréal, P.O. Box 6079, Station Centre-Ville, Montreal, Quebec H3C 3A7 Canada

## Abstract

Photo-initiated chemical vapor deposition (PICVD) has been adapted for use in a jet-assisted fluidized bed configuration, allowing for the encapsulation of magnetic iron oxide nanoparticles on a larger scale than ever reported (5 g). This new methodology leads to a functional coating with a thickness of 1.4–10 nm, confirmed by HRTEM and TGA. XPS and TOF-SIMS characterization confirm that the coating is composed of both aliphatic and polymerized carbon chains, with incorporated organometallic bonds and oxygen-containing moieties. UV-Vis absorbance spectra show that the coating improved dispersion in non-polar solvents, such as n-dodecane. This process represents a first step towards the large-scale, solvent-free post-synthesis processing of nanoparticles to impart a functional coating.

## Introduction

Magnetic iron oxide nanoparticles (MIONPs) are attractive materials for many industrial applications due to their morphology, their chemical composition and magnetic properties (not to mention the high surface to volume ratio common to all nanoparticles)^1–5^. For example, they have been used as (photo-) catalysts to foster chemical reactions^6–10^ and separation processes^11–13^. They have also been employed to improve the mechanical properties of composites and produce electrically conductive materials^14–19^. Of interest, in the biopharmaceutical field, these nanoparticles have been successfully applied to drug delivery, high-contrast magnetic resonance imaging (MRI), stem cell labeling/separation and DNA detection^20–24^.

Organic encapsulation may further improve the use of iron oxide nanoparticles in these applications, by addressing key points^1,25–28^: It may (1) limit their agglomeration; (2) enhance stable particle dispersion via anchor groups with high affinity for the desired dispersion matrix and (3) ease the (bio)conjugation of chemicals/drugs via suitable functional anchor moieties.

Many technical approaches for organic encapsulation have been developed through recent advances in surface engineering. Adsorption of surfactants onto nanomaterials is commonly used to impart colloidal stability, but this approach is severely hindered by thermal instability (suspensions have been shown to lose stability at temperatures as low as 69.4 °C)^29^. Such thermal instability is unacceptable for most areas of nanoparticle use (such as nanofluids or dispersion into polymer matrices). Aqueous co-precipitation of chemical reagents via click chemistry, sol-gel^17,28,30,31^, as well as plasma^32–35^ and thermal chemical vapor deposition^36^ processes are the most common encapsulation techniques to provide a stronger covalent bond. However, in the case of solvent-based reactions, separating coated nanoparticles from solutions often affects process yields drastically (especially if the protocol involves several solvents). While this shortcoming can be curtailed in part when using magnetic nanoparticles, it is generally dodged in the literature for other types of nanomaterials^8,12,28^. Plasma and thermal deposition processes have also important drawbacks, such as high energy consumption, limited scale-up potential (owing to reactor design and low pressure requirements) and compatibility with heat-sensitive substrates^37–39^. In most cases, quantities of coated nanoparticles are limited to micro- or milligrams. Thus, there is an unmet need to develop an industrially viable technique for the encapsulation of nanoparticles. Photo-initiated chemical vapor deposition (PICVD) is well-suited to address this need, as it is a gentle process capable of operating at room temperature and pressure, has a relatively simple reactor design, is capable of using simple precursors (e.g. syngas), consumes low amounts of energy and provides good control over the thin film deposition process^39–42^.

Gas-solid reactors are a popular approach for particles treatment in industry^43–45^. Specifically, fluidized bed reactors are often retained, given that they provide excellent mass and heat transfer (in many cases, this configuration allows for the “stirred-tank” hypothesis to be applied). However, because most nanoparticles belong to to the “Geldart C” group (highly-cohesive)^46,47^, they are not readily fluidized. Indeed, fluidized nanoparticle beds exhibit channeling and form large nano-agglomerates remaining at the bottom of the bed, in turn curtailing bubbling and bed expansion^43,44,47^. The implementation of micro jets, sound waves, impactors and vibrations has been assessed to mitigate/eliminate agglomeration^43,44,48^.

There have been a very limited number of studies involving both particle fluidization and organic encapsulation in a single process^45,49^, and nearly all focused on thermally-based deposition. Zhong *et al*. have been identified as using a photochemical process (photo-initiated cationic polymerization) for encapsulation of potassium chloride (KCl) micro-particles (40–200 mesh) in a fluidized bed^50^. Later on, Pfeffer’s group applied jet-assisted fluidized bed to enhance the fluidization of magnetic iron oxide nanoparticles^51^.

Previously, our group studied the chemistry of syngas photo-initiated chemical vapor deposition (PICVD) on flat surfaces^40,41,52^ and on particulates in a plug-flow configuration^42,53–55^. Herein, we adapt this process for use in a sub-pilot scale jet-assisted fluidized bed reactor (FB-PICVD) to coat large quantities of MIONPs (grams) in order to improve their dispersion and stability in non-polar (hydrophobic) media.

## Results and Discussion

### Physical Characterization

#### Structure and nature of MIONPs

**Figure 1:**
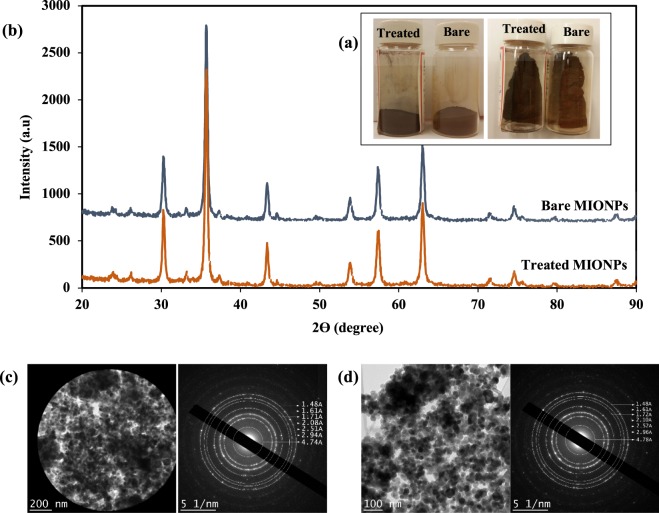
(**a**) MIONPs color before and after treatment, (**b**) X-ray powder diffraction patterns of bare and treated MIONPs, Selected area electron diffraction pattern (SAED) from bare (**c**) and treated (**d**) MIONPs.

MIONPs were treated according to the methodology detailed in section 4.3. Upon particle treatment, we observed a visible color change from light red-brown before treatment to dark black-brown after treatment (Fig. 1(a)). Such a color change in iron oxide could (though unlikely) indicate a crystalline phase change. For further investigation, we conducted X-ray diffraction (XRD) and selected area electron diffraction (SAED) analyses (Fig. 1(b,c) and (d)).

For both bare and treated MIONPs, the main peaks at 30.25°, 35.75°, 43.37°, 53.95°, 57.45°, and 63.09° were observed in the XRD patterns, indicating that the nanoparticles were single phase and there was no alteration in the crystalline structure after treatment. These peaks correspond to the diffraction patterns of either γ-Fe_2_O_3_ or Fe_3_O_4_^56–59^. Considering that both magnetite Fe_3_O_4_ and maghemite γ-Fe_2_O_3_ have a spinel-type crystal structure, it is difficult to distinguish these phases via XRD. SAED analysis confirmed these results: the shape and diameter of the SAED rings in both bare and treated MIONPS indicated that the particles were similar and there was no change in the crystalline structure. The SAED pattern rings can be associate to four compounds: Fe_3_O_4_-magnetite, FeOOH-iron(III) hydroxide, γ-Fe_2_O_3_ -maghemite and α-Fe_2_O_3_ – hematite (least likely)^60^. The nanoparticles retained their magnetic properties after PICVD treatment.

As negative controls, we have performed two series of experiments with the same mass of MIONPs in the FB-PICVD reactor. For the first negative experiments (Negative #1), MIONPs were fluidized with only Ar at 2.4 SLM, with 0.4 SLM of secondary Ar from the micro-jet, in the presence of UVC irradiation (without injection of syngas) for 6 h (i.e. identical to the genuine PICVD treatments). Our second negative control (Negative #2) corresponded to the fluidization of particles with 1.2 SLM of CO and 1.2 SLM of H_2_ (syngas precursor), with 0.4 SLM of secondary Ar, this time without exposure to UVC light. These negative controls were used to independently investigate the effect of UVC lights or syngas on the MIONPs. Supplementary Fig. 1S compares bare particles to their negative control counterparts: no color change or coating were observed for either negative control (Supplementary Table 1S).

#### Coating thickness and thermogravimetric analysis

**Figure 2:**
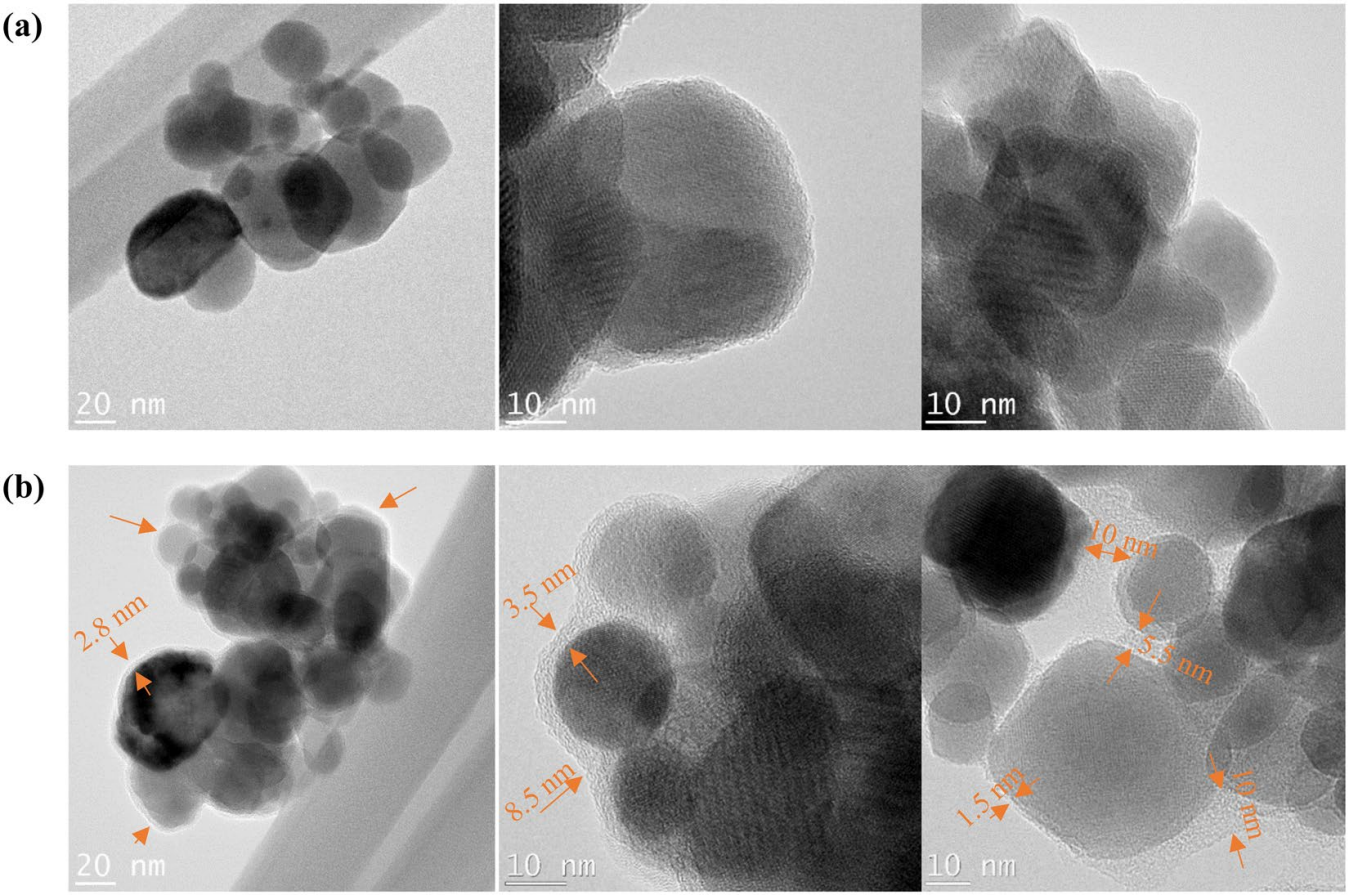
Representative TEM images of (**a**) bare MIONPs and (**b**) treated MIONPs. Red arrows point to coating.

We collected particles dispersed in n-dodecane for TEM analysis (Fig. 2). Bare MIONPs mainly consisted of spherical particles in agglomerated form with a 20–50 nm size distribution; the outermost layer of the particles possessed sharp edges with no other observable phase (Fig. 2(a)). In contrast, the treated particles show a 1.5–10 nm thickness encapsulating layer (Fig. 2(b)). However, fluidization does not appear to impact the agglomeration state of the particles significantly – the coated MIONPs tended to remain in an agglomerated state.

**Figure 3:**
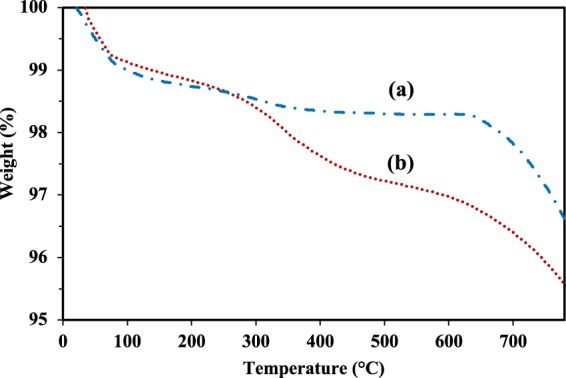
TGA curve of (**a**) bare MIONPs, (**b**) treated MIONPs.

To define the mass content of organic ligands on the surface of MIONPs, thermogravimetric analysis (TGA) was performed on both bare and treated particles (Fig. 3), showing four phases of weight loss:

1. 20–75 °C: Both bare and treated MIONPs showed ~1% weight loss. This was attributed to adsorbed humidity;
2. 75–300 °C: Both bare and treated MIONPs showed ~0.5% weight loss, which was attributed to chemisorbed humidity;
3. 300–600 °C: Treated particles showed a weight loss of ~2%, bare particles only lost ~0.5%. This corresponds to the pyrolysis of organic content, with a carbon content 4-times higher in the treated MIONPs compared to the bare particles, which can therefore be attributed to PICVD treatment. Note that bare MIONPs do contain a small amount of carbon attributed to their production process (organic phase thermal decomposition of Fe(acetylacetonate) or iron pentacarbonyl Fe(CO)_5,_ followed by oxidation^5,61^).
4. 600–800 °C: Both bare and treated MIONPs showed ~1.7% and ~1.5% weight loss, respectively. This is attributed to the organic content from the MIONPs production process^5,61^ (i.e. not associated with PICVD treatment).

We used ImageJ software to determine the average thickness of the coating from the TEM images. We observed an average thickness of 4.5 nm for a 6 h treatment, which corresponds to roughly 0.75 nm coating per hour of treatment. On the other hand, the total weight loss corresponding to the treatment process (TGA decomposition from 300 to 600 °C) corresponds to 0.18 mg for the 6 h treatment. Thus, the mass deposition rate would be 0.03 mg/h, or in another word every 0.03 mg of deposit is equal to 0.75 nm in thickness. Therefore, assuming average particle diameter size of 25 nm, the density of deposit roughly would be 4.5 × 10^−2^ g/cm^3^, significantly lower than the density reported for plasma polymer coatings (0.65 g/cm^3 ^^61^). This hints at the fact that either not all particles are coated due to circulation issues (see section 2.3), or that the coating is strictly deposited on agglomerates (to obtain the average 4.5 nm coating with a comparable density to plasma polymers, agglomerates would have to be on the order of 150 nm).

### Chemical Characterization

**Figure 4:**
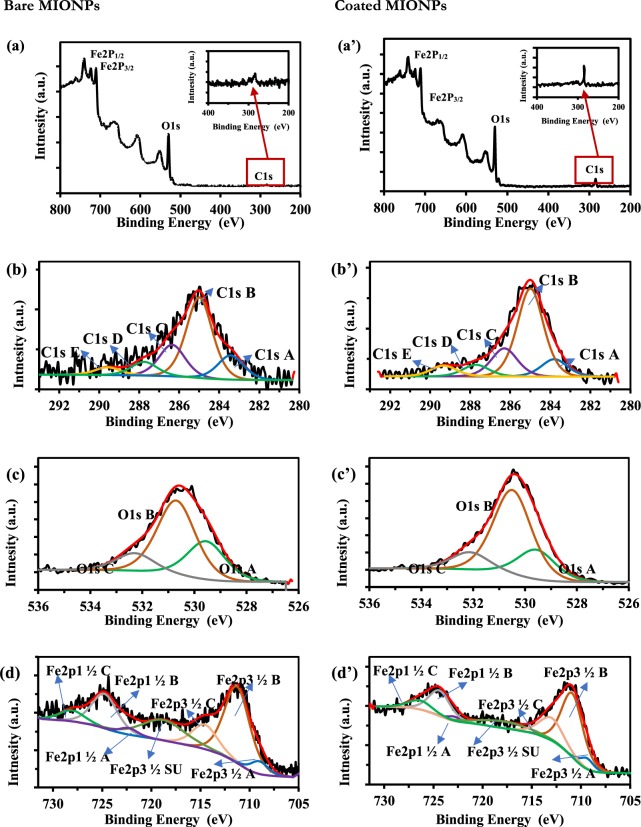
XPS Surveys spectra (**a**) bare MIONPs, (a’) treated MIONPs; deconvolutions of high-resolution spectra of C1s for (**b**) bare MIONPs, (b’) treated MIONPs; High resolution oxygen peak deconvolution for (**c**) bare MIONPs and (c’) treated MIONPS; High resolution iron peak deconvolution for (**d**) bare MIONPs and (d’) treated MIONPS.

With the encapsulation of MIONPs by PICVD confirmed, we wanted to know if the deposited film possessed the same chemistry as the thin films deposited on flat silicon wafers from our previous work^41^. XPS characterization provided further information on the deposited oligomeric film (Fig. 4) for MIONPs treated with PICVD. The survey spectrum for treated particles (Fig. 4(a’)) showed an increase in carbon content, as deduced from the C1s peak compared to bare particles (Fig. 4(a)). This is in agreement with the TEM micrographs and TGA analysis demonstrating the formation of an organic film on MIONPs due to the syngas PICVD process. Deconvolution of the C1s, O1s and Fe2p3 high-resolution spectra allowed for a more detailed characterization of MIONPs (Table 1)^62^.

**Table 1:** Deconvolutions of high-resolution spectra and their respective atomic % (source of BE obtained by comparison to ref.^62^).

Peak Name	Centered Binding Energy [eV]	Possible Structure (1)	Atomic % of MIONPs	
			Bare	Treated
C1s A	283.4	Fe–*C	2.1	2.1
C1s B	285.0	*C–*C, * C–H, *C=*C, etc.	6.8	11.1
C1s C	286.4	*C–O–*C, *C–O–Fe, *C–O–H	2.7	3.5
C1s D	287.8	O=*C–C, O–C–O	1.2	1.4
C1s E	289.6	O=*C–O, O=*C–O–Fe	0.7	1.3
Fe2p3^1/2^ A	709.0	C–Fe(O)–C, C–Fe(O)–O	2.2	1.4
Fe2p3^1/2^ B	711.3	Fe^+3^ in iron oxide	19.0	13.6
Fe2p3^1/2^ C	714.6		6.1	5.4
Fe2p3^1/2^ SU	718.8		8.6	3.4
O1s A	529.6	Fe–*O	14.8	12.8
O1s B	530.7	H–*O–Fe, *O=C	28.9	36.5
O1s C	532.3	C–O–*Fe, *O=C–*O–Fe	6.9	7.5

Bare MIONPs consisted mainly of iron and oxygen, though high-resolution spectra also showed carbon - C1s (Fig. 4(b)). A sub peak at BE ≅ 283.4 eV indicated covalent bonding between Fe and C (~2%At., Table 1), with a correspond sub peak in the Fe2p3^1/2^ spectrum (A, BE ≅ 709.0 eV, in Fig. 4(d)). Most of the carbon present in the C1s spectrum was oberved at BE ≅ 285.0 eV and 286.4 eV (Fig. 4(b), C1s B and C1s C), assigned to C–C and C–H bonds and C–O–C bonds, respectively. The remaining carbon content is in the form of O=*C–C, O–C–O or O=*C–O–Fe as deduced from sub peaks at BE ≅ 287.8 eV and 289.6 eV (Fig. 4(b), C1s D and C1s E). As stated previously, the presence of carbon in bare MIONPs is attributable to their production process^5,61^.

The iron spectrum sub peaks at BE ≅ 711.3 eV, 714.6 eV and 718.8 eV (B, C, SU, respectively) as well as their corresponding Fe2p1^1/2^ sub peaks correspond to Fe^+3^ in iron oxide (Fig. 4(d)). These sub peaks are consistent with the oxygen bonds indicated as O1s A, O1s B and O1s C at BE ≅ 529.6 eV, 530.7 eV and 532.3 eV (Fig. 4(c)).

Treated MIONPs were characterized by a 6.2%At. and 5.9%At. increase in the O1s and C1s peaks, respectively, and a 12.1% decrease in Fe. This observation is consistent with the MIONPs being covered by an oligomeric film composed of C and O – also leading to a Fe peak attenuation. C1s high resolution peak deconvolution indicated that most of the carbon deposition is in the form of C–C, C=C and C–H bonds at BE ≅ 285.0 eV and 286.4 eV (Fig. 4(b’), C1s B and C1s C), with slight differences for other types of carbon content. O1s peak deconvolution indicated that the main increase of O sub peaks was in the form of H–*O–Fe and *O=C (Fig. 4(c’), O1s B). This agrees with our previously published reports where we demonstrated that syngas PICVD deposition on flat silicon formed a film of aliphatic hydrocarbons bonded to oxygen. However, we also previously showed that films deposited through syngas PICVD consist of C, O and Fe, owing to the presence of Fe(CO)_5_ in the CO gas stream^41^. This Fe content in the coating is difficult to distinguish here from the Fe in the substrate (Fe_2_O_3_). To confirm its presence, we can use the Beer-Lambert law:

$${I}_{z}={I}_{0}\,{e}^{-\frac{z}{\lambda cos\theta }}$$

Which tells us that the XPS signal intensity from a layer of atoms at distance *z* (*I*_*z*_) is less than the signal intensity obtained from the layer at the original surface *I*_0_ by a factor of ${e}^{-\frac{z}{\lambda cos\theta }}$ (*λ* is the photoelectron attenuation length and *θ* is the photoemission angle). If we consider a minimum coating thickness *z* of 3 nm and assume an attenuation length of *λ* of 3.9 nm (calculated by the XPS software), and Ɵ = 0° *I*_*z*_/*I*_0_ should ≅0.46. However, in our case, we have (based on Table 1):

$$\frac{{I}_{z}}{{I}_{0}}=\frac{At \% \,of\,\mbox{''}\mathrm{Fe}\mbox{''}\,in\,treated\,MIONPs}{At \% \,of\,\mbox{''}\mathrm{Fe}\mbox{''}\,in\,bare\,MIONPs}=\frac{23.8}{35.9}=0.66$$

This amount is higher than expected, highly suggesting that there is also some iron deposition coming from the PICVD process itself, in agreement with previous reports.

These XPS analyses have demonstrated that the oligomeric films deposited on nanoparticles are similar in composition to the films deposited on previously studied flat substrates^41^. Specifically, both oligomeric films consist of organometallic moieties and light hydrocarbons, with a similar distribution as a function of BE (for example, 0.8%At at 286.4 eV for nanoparticles, compared to 1.2% At for flat substrates).

Further chemical characterizations using TOF-SIMS and ATR-FTIR have been performed to confirm the XPS findings. These are presented in Supplementary Figs 2S, 3S and 4S and Table 2S.

### Processing considerations

Knowing that the coating chemistry is comparable to that of syngas PICVD coatings generated on flat substrates, it is possible to compare the present results with our previous studies in order to gain processing insight. In Farhanian *et al*.^41^, we showed that film thickness varies both as a function of treatment time and gas residence time within the reactor. Residence time (τ) is defined as the reactor volume divided by the volumetric flow rate. For the range of residence time of interest, in the same system (for a 2 hour treatment), we can consider a linear relation between residence time and film thickness (d) based on previous data^40^: d = 165τ. Considering the dimensions of the FB-PICVD reactor (D = 2.5 cm, h=1.8 m) and the total gas flow rate (Q = 2.8 SLM), the residence time is 0.3 min. By applying the linear relation, we therefore expect a film thickness of 49.5 nm after 2 h (i.e. a deposition rate of 0.41 nm/min). However, after 6 h, the film thickness on nanoparticles is only 4.5 nm (section 3.1.2), corresponding to a deposition rate of 0.01 nm/min. This difference can be attributed to the masking effect caused by the nanoparticles during fluidization: only particles near the quartz wall are exposed to UVC irradiation. Interestingly, based on the expected deposition rate (0.41 nm/min), 11 min would be required to reach the observed coating thickness of 4.5 nm: in other words, each particle was in proximity to the quartz wall during treatment only 3% of the time.

This indicates that the dimensions and fluidization parameters of the FB-PICVD reactor are not optimal. Indeed, we have attempted brief experiments at higher particle loadings (9 g – the upper limit of our current reactor configuration for this particular type of NP): fluidization was poor and the masking effect was accentuated (particle circulation to the quartz wall is further decreased) (data not shown). To improve the efficiency of the process (and therefore decrease treatment time), various methods should be considered: decreasing the reactor diameter, increasing precursor residence time, improving particle circulation through other assisted fluidization techniques (beyond a single micro-jet)^44,63^. The FB-PICVD reactor design could be further improved through the use of a jet-impactor system^48^ to reduce the formation of agglomerates. These process improvements are the focus of on-going work.

### Dispersion

**Figure 5:**
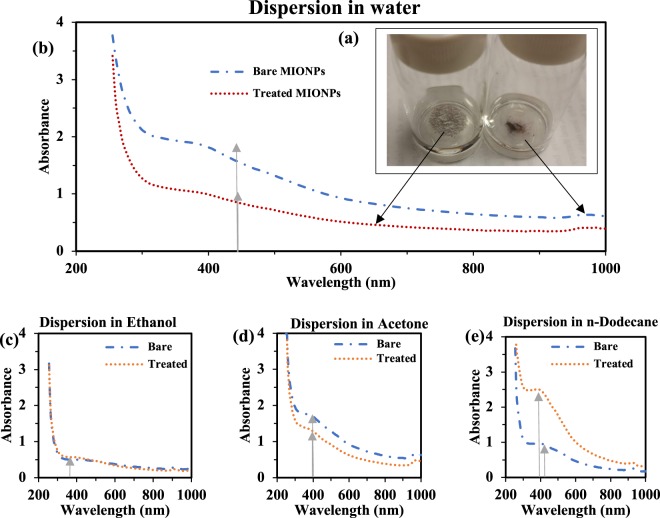
(**a**) Photograph of bare and treated MIONPs dispersed in DI water, UV-Vis absorbance spectrum of bare (blue line) and treated (red dot) MIONPs in (**b**) Water, (**c**) Ethanol, (**d**) Acetone, (**e**) n-Dodecane.

Despite the processing limitations, the functional coating deposited had a significant effect on the dispersion of MIONPs in many solvents. Before any treatment, bare particles tended to settle in water (Fig. 5(a)-right). In stark contrast, after treatment, the particles remained at the water surface (Fig. 5(a)-left). When sonicated, treated particles tended to move back to the surface after several hours. To quantify the dispersion of coated MIONPs in various solvents, we used UV-Vis spectroscopy. Indeed, the peak absorption wavelength (λ_max_) of the particles is highly dependent on their dispersion media^64,65^. Any shift in λ_max_ or change in absorbance at a fixed λ can thus be used to detect changes in the surface composition of the nanoparticles^64,65^. Figure 5(b) shows UV-Vis absorbance of bare and treated MIONPs dispersed and sonicated in water. For treated particles, we observed decreased absorbance, highly suggesting that the coating of our MIONPs is hydrophobic. That is, the coated MIONPs did not remain in suspension – they accumulated at the surface in order to minimize their interaction with water molecules.

To gain additional insight into the MIONP surface composition, we dispersed and sonicated both bare and treated MIONPs in different solvents such as ethanol (polar-protic with polarity index = 0.66), acetone (polar-aprotic with polarity index = 0.35), and n-dodecane (non-polar) (Fig. 5(c,d) and (e), respectively).

In ethanol, there was no change in absorbance, while there was a slight change in the case of acetone. In both polar solvents, no shifts in the peak wavelength (λ_max_) occurred. On the other hand, dispersion of our coated particles in non-polar n-dodecane not only increased absorbance (indicating that coated particles dispersed more readily), but shifted λ_max_ from roughly 430 nm to 400 nm^64,65^. Considering the fact that the optical properties of metal nanoparticles depend strongly on their shape, size, absorbed species on their surface as well as interactions between the particles^66^, this shift in λ_max_ can be attributed to (1) shape and size changes due to coating or (2) different UV absorption properties of conjugated thin films^67,68^. Upon addition of a thin layer at the surface of MIONPs, the outer layer of bare nanoparticle is masked. Thus, due to a chemistry change of the outer layer, interaction between the MIONPs and the oligomeric film may change the refractive index, which in turn explains the shift in λ_max._ This has been observed in the past for the surface modification of iron oxide nanoparticles using polyethylene glycol (PEG) surfactants of various lengths, with longer chain lengths leading to a higher wavelength shift^69^. Similar results were observed for Fe_3_O_4_ nanoparticles coated with polyacrylic acid (PAA) and polystyrene (PS)^70^. Moreover, the size and shape of the nanoparticle agglomerates, influenced by the polarity of the solvent in which they are dispersed, can change their surface plasmon resonance (SPR), contributing to in-plane dipole resonance (longitudinal) or out-of-plane dipole resonance (transverse) and leading to a shift in λ_max_ (this is typically observed in the case of encapsulated silver nanoparticles^66^ but there can be a minor effect for iron as well^65^).

## Conclusions

FB-PICVD, a scalable process for the encapsulation of nanoparticles, has been demonstrated using syngas as a functionalization precursor. Magnetic iron oxide nanoparticles (MIONPs) were selected as encapsulation substrates, given their wide range of applications that could benefit from an organic coating. Despite some minor processing limitations, 5 g of nanoparticles were treated, far beyond the typical amount of particles treated (micrograms or milligrams). The syngas PICVD leads to a color change of the MIONPs, demonstrated to be directly attributable to the functional coating (no change in crystallinity). TEM micrographs confirmed the presence of a coating on the nanoparticles (and their agglomerates), on the order of 1.4 to 10 nm, corroborated by an increased amount of carbon observed by TGA. XPS analysis demonstrated that the chemistry of the coating is not dissimilar to films deposited on a flat substrate, showing a 6% increase in carbon content of particles after PICVD treatment, and a functional coating consisting mainly of aliphatic hydrocarbons (e.g. –CH, –CH_2_, –CH_3_) and polymerized chains (e.g. C≡C, C=C, C–C) (4.3%), as well as ketones, esters and carbonates (0.2–0.8%). The functional film had an impact on dispersion properties, with UV-Vis spectroscopy showing evidence of a hydrophobic deposit that enhanced dispersion in non-polar solvents like n-dodecane. In order to accurately control the thickness of the coating, fluidization of the nanoparticles in the fluidized bed reactor will need to be improved, namely to control the formation of agglomerates and ensure uniform circulation of the particles. On-going work in our laboratory focuses on improving the treatment process via assisted techniques such as pulsed fluidization, jet-impactor assemblies and vibration (or a combination thereof), as well as targeting alternate reaction precursors that could be used to promote dispersion into polar media. This paves the way towards scale-up to industrially relevant levels, namely, moving to continuous processing with the help of a nanoparticle feeder system.

## Methods

### Materials

Spherical magnetic iron oxide nanoparticles (MIONPs) with 20–30 nm diameter (Fe_2_O_3_, gamma with 98% purity and density = 5.24 g/cm³ at 20 °C) were purchased from NanoAmor Inc. Argon gas with a purity of 99.998%, carbon monoxide and hydrogen gases (the components of syngas) with a purity of 99.8% were purchased from Air Liquide Canada. Solvents such as ethanol, n-dodecane, and acetone with 99.9% purity were purchased from Fisher Scientific Co.

### Experimental set-up

**Figure 6:**
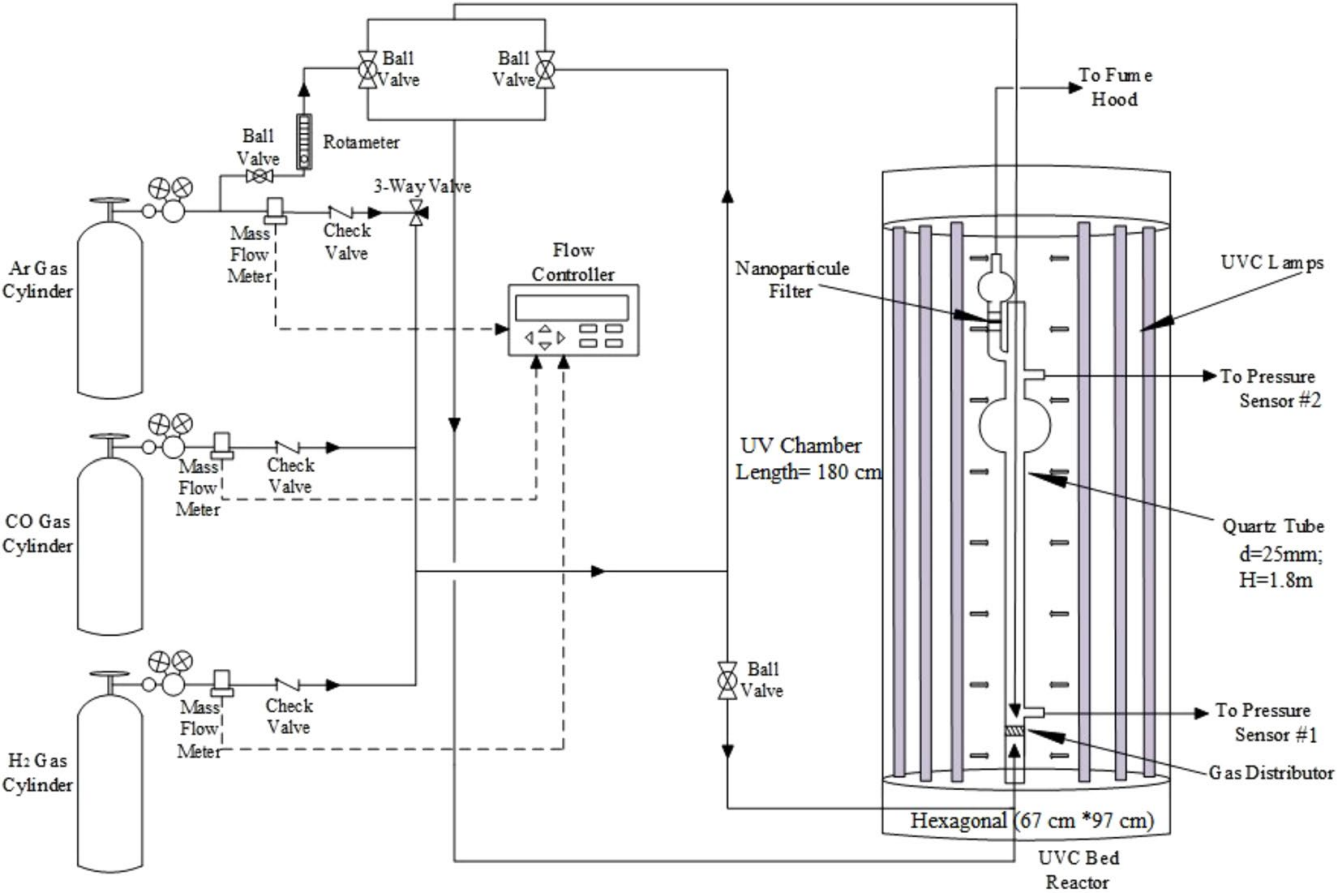
Schematic diagram of the FB-PICVD experimental setup.

The fluidized bed photo-initiated chemical vapor deposition (FB-PICVD) process was carried out in a custom-made fluidized bed reactor (Montreal Glass Blowing) which consisted of a quartz column with a 25-mm internal diameter, a 28.8-mm external diameter and 1.8-m height (Fig. 6). The length of column was modular and connections were 24/40 male/female taper joints. A fritted disc with a pore size of 15–40 microns was mounted at the bottom of the column as a gas distributor. An expansion section as well as a pre-filter (i.e., another fritted disc with the same pore size) were installed at the outlet of the reactor to retain any entrained particles. The outlet gas flow then passed through a HEPA filter to remove any further particles before going to the ventilation system.

There were two pressure measurement ports; one was located right above the distributor and the other just before the pre-filter at the top of the column. These pressure ports were connected to a digital manometer (Series 475 Mark III, Dwyer Instruments, 0 to 50 kPa). The reactor column was also equipped with a 1/8” stainless steel tube compressed to form a 100-µm nozzle, to be used as a micro-jet to promote fluidization and provide secondary flow. This micro-jet nozzle was located at the center of the column pointing downwards at about 0.5 cm above the gas distributor. Elastomer-sealed thermal mass flow controllers (SLA5850S Brooks, Polycontrols) connected to a 4-channel electronic control module and power supply were used to supply the inert and reactive precursor gas streams to the system. Secondary flow through the micro-jet nozzle was controlled via a calibrated low flow rotameter (McMaster-Carr. Co.).

The fluidized bed was housed in a custom-made UV cabinet (Daavlin Co.) (Fig. 6S) consisting of 28 UVC lamps. These lamps had a main emission peak at a wavelength of 253.7 nm and a light intensity of 0.012 W/cm^2^ at the reactor distance of 33 cm. The wavelength and light intensity were measured via an Ocean Optics Spectrometer/USB4000-XR1-ES and an ILT1700 Research Radiometer coupled with a SED240/QNDS2/ W254 nm sensor (International Light Technologies), respectively.

### Fluidization and encapsulation process

5.0 g of vacuum-stored MIONPs were loaded into the reactor (Fig. 6). The UVC lamps were turned on for 30 min prior the experiments. Then, 2.4 SLM argon gas (Ar) was injected gradually to fluidize the particles, and facilitate the fluidization of particles by removing adsorbed humidity while evacuating the oxygen from the reactor.

To launch the encapsulation process, hydrogen (H_2_) and carbon monoxide (CO) were fed through the reactor distributor gradually, decreasing the Ar flow rate proportionally to maintain particle fluidization. Once the syngas (CO + H_2_) flow reached its set point, the Ar micro-jet was activated and adjusted to 0.4 SLM. The minimum fluidization velocity (U_mf_) was determined by measuring the pressure drop across the particle bed as a function of the syngas superficial gas velocity (U). Supplementary Fig. 5S shows this differential pressure drop as well as non-dimensional bed expansion as a function of superficial gas velocity during micro-jet assisted fluidization. Considering these curves, U_mf_ was 5.8 cm/s; however, to improve flowability of particles and increase bed expansion we targeted U = 8.5 cm/s (CO and H_2_ flows set to 1.2 SLM – limit of the system), with a micro-jet flow of 0.4 SLM. This fluidization velocity was retained as it was the highest gas flow that could be employed in the system that both allowed for a bubbling regime (greater mixing) and prevented excessive entrainment. The micro-jet Ar flow rate was intentionally kept low to avoid dilution of the reactive precursors and avoid excessive gas consumption. The UVC lamps provided sufficient energy to excite the precursors, which would lead to the formation of an oligomeric film on the particles^40,41^. Total treatment time was 6 h, and the film generation reaction was terminated by turning off the UVC lamps and switching the gas flow back to inert gas in order to flush the reactor. Over the course of a typical deposition experiment, the temperature increased from room temperature (~20 °C) to approximately 63 °C; the outlet pressure was atmospheric.

### Characterization

To assess the impact of PICVD treatment, if any, on the crystalline phases present in the MIONPs, the particles were analyzed using X-ray diffraction (Philipps X’Pert XRD system) with a Cu anode (K = 0.15406 nm) at a voltage of 50 kV and a current of 40 mA, at room temperature. Scans were conducted from 20.01 to 89.99° at a step size of 0.02° 2Ɵ/min. Particles phases were identified using the Joint Committee on Powder Diffraction Standards (JCPDS) and ICDD-powder diffraction data system (File 39–1346)^58^.

A UV-Visible spectrometer (OceanOptics USB4000-UV-VIS) was used to define the state of aggregation of the nanoparticles in various solvents. The absorption spectrum of the suspensions was obtained from 200 to 1000 nm.

The amount of grafted oligomeric film was determined using thermogravimetric analysis (TGA-METTLER TOLEDO apparatus) operating from 25 to 800 °C at a heating rate of 10 °C/min under nitrogen gas flow. The analyses were conducted on pellets (14.0 ± 2.5 mg) of both bare and treated particles, to ensure that any mass difference recorded was due to thermal decomposition and not loss of particles in the machine.

Transmission electron microscopy (TEM-JEOL JEM-2100F) was applied to determine the thickness of the coatings. The TEM was operated at 200 kV to acquire bright field images of the samples. To prepare samples for TEM analysis, particles were dispersed and sonicated in a solvent (methanol, ethanol or n-dodecane). The TEM grids (D20040 grids with formvar lacey carbon, mesh 400, Cu metal SOQUELEC International) were soaked in these suspensions for a few seconds and dried at room temperature. This was done to improve the uniformity of dispersion on the TEM grids (as opposed to simply depositing the particles onto the grids).

X-ray photoelectron spectroscopy (XPS) characterization was carried out using a VG ESCALAB 3 MKII system equipped with a non-monochromatic Mg-Kα radiation source, operated at 300 W (15 kV, 20 mA). The pass energy of the analyzer was fixed to 100 eV for survey scans and 20 eV for high-resolution scans in 1.00 eV and 0.05 eV increments, respectively. An X-ray incident angle of 15° with a corresponding penetration depth of ~10 nm was used for survey scans. The binding energy scale of the system and charging of the samples was corrected and calibrated using C1s at 285.0 eV for both high resolution and survey analyses. The analyzed window surface was 2 × 3 mm and the base pressure of the UHV analysis chamber was kept under 5 × 10^−9^ Torr. For all spectra, a Shirley background was used before fitting the peaks with a symmetric Gauss-Lorentz sum function of 50% ratio (0%: pure Gauss, 100%: pure Lorentz). For metallic elements, in this case Fe, a Smart background was selected instead of classic Shirley background. For all peaks, a full width at half-maximum (FWHM) of 1.6, 1.8 and 2.7 eV for C, O, and Fe were used respectively. Moreover, the atomic ratios of elements in the samples were calculated according to the corresponding fitted peak area and corrected by the instrument sensitivity factors. The Scofield sensitivity factors modified for the instrument were 0.25, 0.66 and 2 for C, O and Fe respectively. In this manuscript, all peaks are reported by their centered binding energy (BE). Avantage XPS analysis software was used to deconvolute the peaks in the high-resolution spectra and calculate the peak area.

## Electronic supplementary material

Supplementary information

## Data Availability

All pertinent data is presented within the manuscript. Raw data can be provided upon request as needed.
