# Supplementary material for: Large-Scale Encapsulation of Magnetic Iron Oxide Nanoparticles via Syngas Photo-Initiated Chemical Vapor Deposition
Source: Sci Rep. 2018 Aug 15;8:12223. doi: 10.1038/s41598-018-30802-1 (PMC6093859; doi:10.1038/s41598-018-30802-1)
Supplement: Supplementary file 1 — Supplementary information [file 41598_2018_30802_MOESM1_ESM.docx]

Large-Scale Encapsulation of Magnetic Iron Oxide Nanoparticles via Syngas Photo-Initiated Chemical Vapor Deposition

Donya Farhanian ^a^, Gregory De Crescenzo ^a^, Jason R. Tavares* ^a^

^a^ CREPEC, Department of Chemical Engineering, École Polytechnique de Montréal, P.O. Box 6079, Station Centre-Ville, Montreal, Quebec, H3C 3A7, Canada.

*Corresponding author. Tel.: +1 514 340 4711 Ext. 2326; fax: +1 514 340 4159. E-mail address: jason.tavares@polymtl.ca (J.R. Tavares).

**Control experiment results:**

| **(c)**  **(c)**  **(b)**  **(b)**  **(a)**  **(a)** 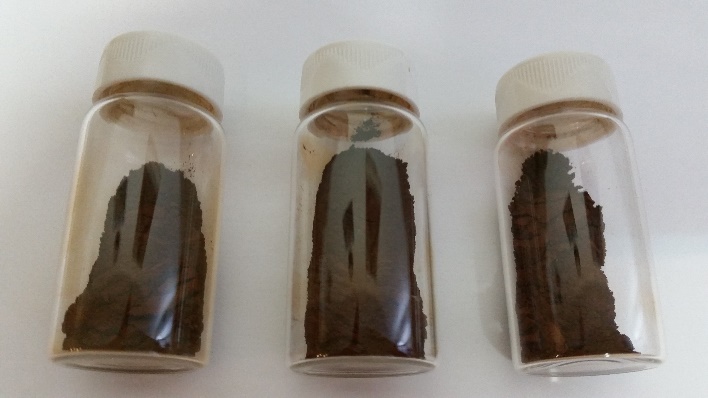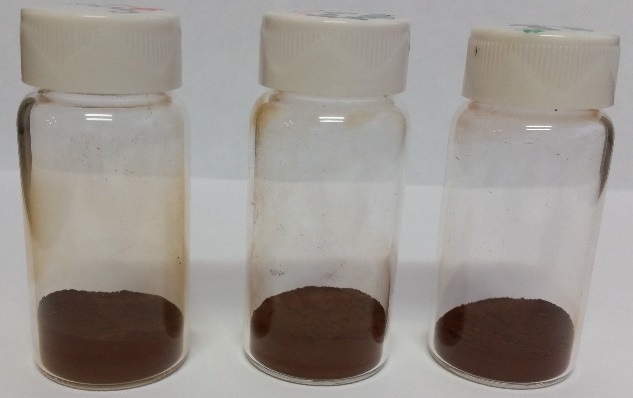 | | |
| --- | --- | --- |
| 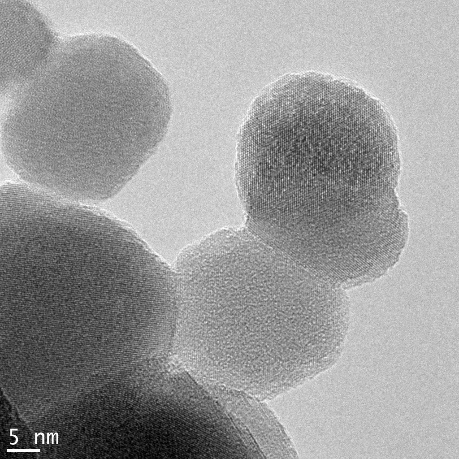 | **(b)** 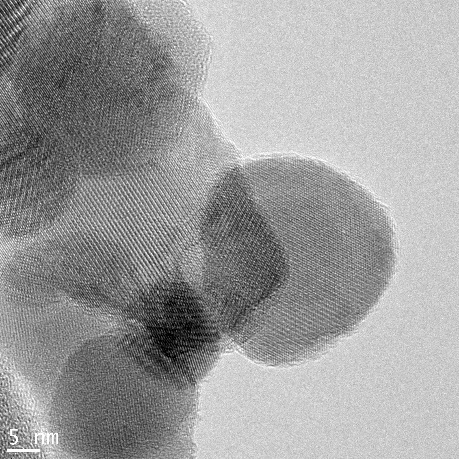 | **(c)** 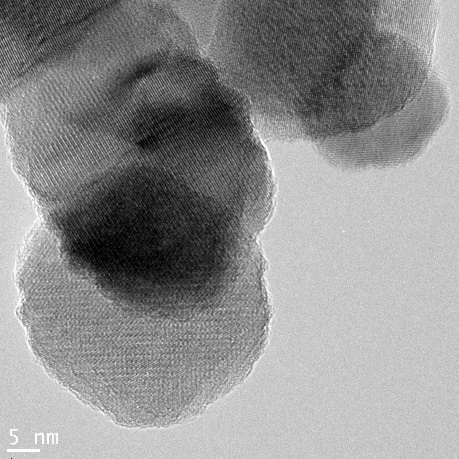 |
| Figure 1S. Representative picture and TEM images of (a) Bare MIONPs, (b) Negative #1: MIONPs under UVC and argon gas treatment, (c) Negative #2: MIONPs under syngas precursors and argon gas treatment.  **(a)** | | |

| Table 1S. Elemental At.% of bare and treated MIONPs in negative control experiments. |
| --- |
| \| **Name** \| **Peak BE** \| **At. %** \| \| \| \| --- \| --- \| --- \| --- \| --- \| \| **Bare** \| **Negative #1** \| **Negative #2** \| \| **C1s** \| 285.0 \| 13.5 \| 13.2 \| 14.4 \| \| **O1s** \| 530.0 \| 50.6 \| 57.4 \| 55.3 \| \| **Fe2p3** \| 711.4 \| 35.9 \| 29.4 \| 30.3 \| |

**Additional functional group analysis**

For further chemical characterization of both bare and coated MIONPs, we used static time-of-flight secondary ion mass spectrometry (TOF-SIMS) analysis. This analysis was carried out with a ION-TOF SIMS IV (from ION-TOF GmbH, Germany) equipped with a 25 keV Bi^3^ liquid metal ion source as the primary ion beam source, in high current bunched mode. A bunching system gives pulse durations of 21.9 ns with a mass resolution M/ΔM better than 8,000 around m/z = 100 in positive and negative SIMS modes. Depth profiling (10 Ǻ maximum) was performed in non-interlaced mode, where the analysis and sputtering occurred with a 50 μm × 50 μm analysis area confined within a 500 μm × 500 μm sputter area, keeping the total dose below 5 × 10^12^ ions cm^-2^ (so called static conditions). Three measurements at adjacent spots were performed on the samples and positive and negative ion spectra were compared to confirm uniformity. Ion spectra were internally calibrated using H^+^, H_2_^+^, CH_3_^+^, C_2_H_2_^+^, C_3_H_5_^+^ and H^−^, C^−^, CH^−^, C_2_H^−^, and C_4_H^−^peaks, respectively.

Figure 3S compares negative and positive ions in both bare and coated samples. Clearly the amounts of negative and positive ions is higher in coated MIONPs compared to bare MIONPs, in agreement with XPS results. Table 2S shows some of the negative and positive ions representing each mass up to Mass = 100 u. Moreover, Figure 4S shows the zoomed region showing two groups in these samples.

| **(a)** | 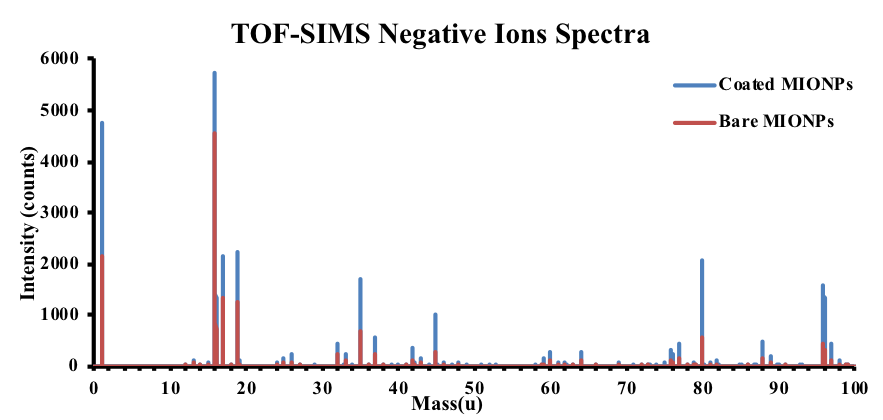 |
| --- | --- |
| **(b)** | 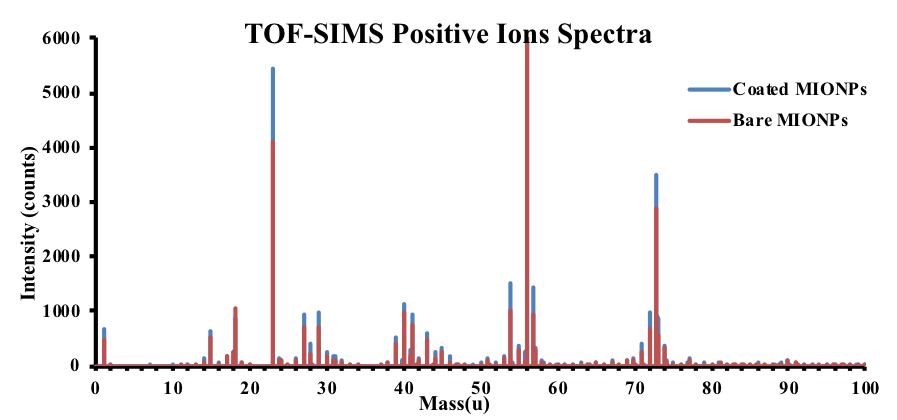 |
| Figure 2S. TOF-SIMS analysis of bare and coated MIONPs. (a) negative ion spectra, (b) positive ion spectra | |

| Table 2S. TOF-SIMS negative and positive ion assignment |
| --- |
| \| **Negative Ions** \| \| **Positive Ions** \| \| \| \| \| \| \| --- \| --- \| --- \| --- \| --- \| --- \| --- \| --- \| \| **Center Mass (u)** \| **Ion Assignment** \| **Center Mass (u)** \| **Ion Assignment** \| **Center Mass (u)** \| \| **Ion Assignment** \| \| \| 1 \| H^-^ \| 26 \| C_2_H_2_^+^ \| 77 \| \| C_6_H_5_^+^ \| \| \| 24 \| C_2_^-^ \| 27 \| C_2_H_3_^+^ \| 78 \| \| C_6_H_6_^+^ \| \| \| 37 \| C_3_H^-^ \| 29 \| C_2_H_5_^+^ \| 81 \| \| FeC_2_H^+^ \| \| \| 45 \| COOH^-^ \| 39 \| C_3_H_3_^+^ \| 82 \| \| C_2_H_2_Fe^+^ \| \| \| 49 \| C_4_H^-^ \| 43 \| C_3_H_7_^+^ \| 83 \| \| FeC_2_H_3_^+^ \| \| \| 56 \| Fe^-^ \| 56 \| Fe^+^ \| 84 \| \| COFe^+^ \| \| \| 57 \| FeH^-^ \| 57 \| FeH^+^ \| 85 \| \| FeCOH^+^ \| \| \| 71 \| C_3_H_3_O_2_^-^ \| 57 \| C_4_H_9_^+^ \| 86 \| \| CH_2_OFe^+^ \| \| \| 72 \| FeO^-^ \| 65 \| C_5_H_5_^+^ \| 87 \| \| FeCOH_3_^+^ \| \| \| 73 \| FeOH^-^ \| 68 \| CFe^+^ \| 91 \| \| C_7_H_7_^+^ \| \| \| 75 \| C_2_H_3_O_3_^-^ \| 69 \| FeCH^+^ \| 101 \| \| FeCOOH^+^ \| \| \| 85 \| C_7_H^-^ \| 69 \| C_5_H_9_^+^ \|  \|  \| \| \| 88 \| FeO_2_^-^ \| 70 \| CH_2_Fe^+^ \|  \| \|  \| \| \| 92 \| C_3_Fe^-^ \| 71 \| FeCH_3_^+^ \|  \| \|  \| \| \| 93 \| C_6_H_5_O^-^ \| 72 \| FeO^+^ \|  \| \|  \| \| \| 100 \| CO_2_Fe^-^ \| 73 \| FeOH^+^ \|  \| \|  \| \| |

| **(a)** | **Coated MIONPS**  **Bare MIONPS** 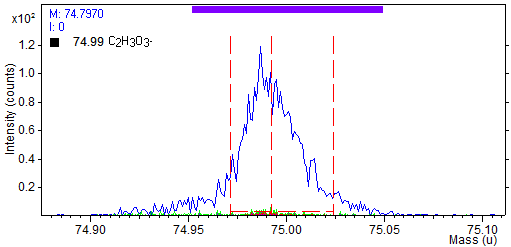 |
| --- | --- |
| **(b)** | **Coated MIONPS**  **Bare MIONPS** 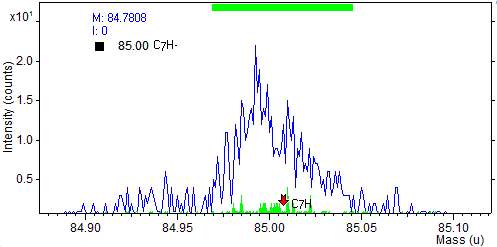 |
| Figure 3S. TOF-SIMS analysis of bare and coated MIONPS: (a) C_2_H_3_O_3_- with mass of 75 representing carbonyl groups and (b) C_7_H with mass of 85 representing linear aliphatic groups. | |

Moreover, in order to define the chemical groups present in the deposited film, we prepared a KBr pellet and treated the sample for 120 min with 0.2 L/min of both CO and H_2_ using PICVD. Attenuated Total Reflection Fourier Transform Infrared Spectroscopy (ATR-FTIR) has been used for chemical analysis of the samples on these KBr coated disks using a Perkin Elmer 65 FTIR-ATR instrument. FTIR spectra were recorded over a wavenumber range of 650-4000 cm^−1^ (total of 128 accumulated scans) and a 4 cm^−1^ resolution. Figure 5S shows the ATR-FTIR spectra and their corresponding chemical groups.

| 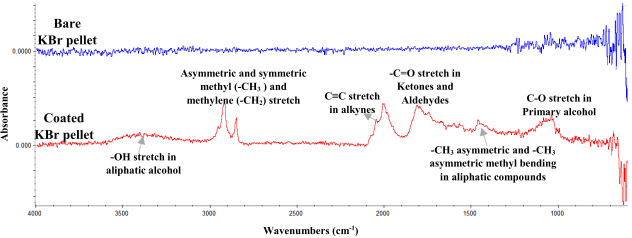 |
| --- |
| Figure 4S. ATR-FTIR spectra of bare and coated KBr samples |

**Fluidization and defluidization of MIONPs**

| **(a)** 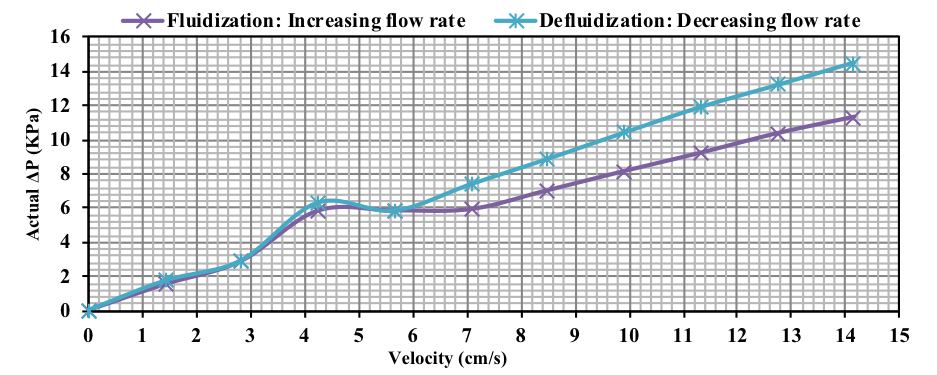 |
| --- |
| **(b)** 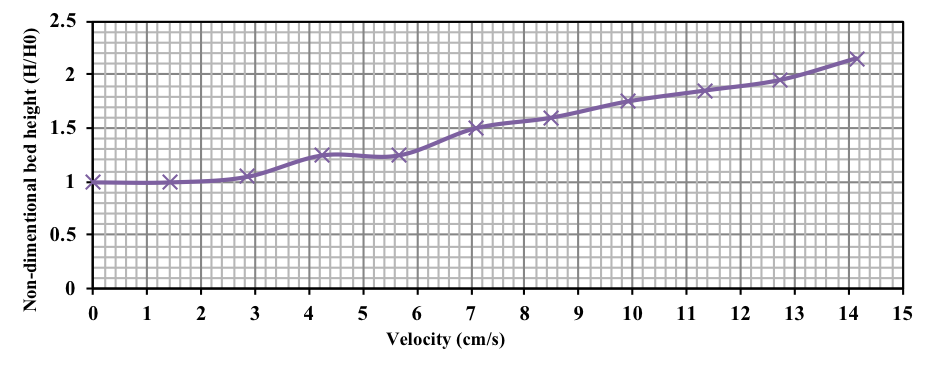 |
| Figure 5S. (a) The actual pressure drop as a function of gas velocity during ﬂuidization (increasing flow rate) and defluidization (decreasing flow rate) of MIONPs, (b) The non-dimensional ﬂuidized bed height as a function of gas velocity for MIONPs. |

**Picture of operating experimental set-up**

| 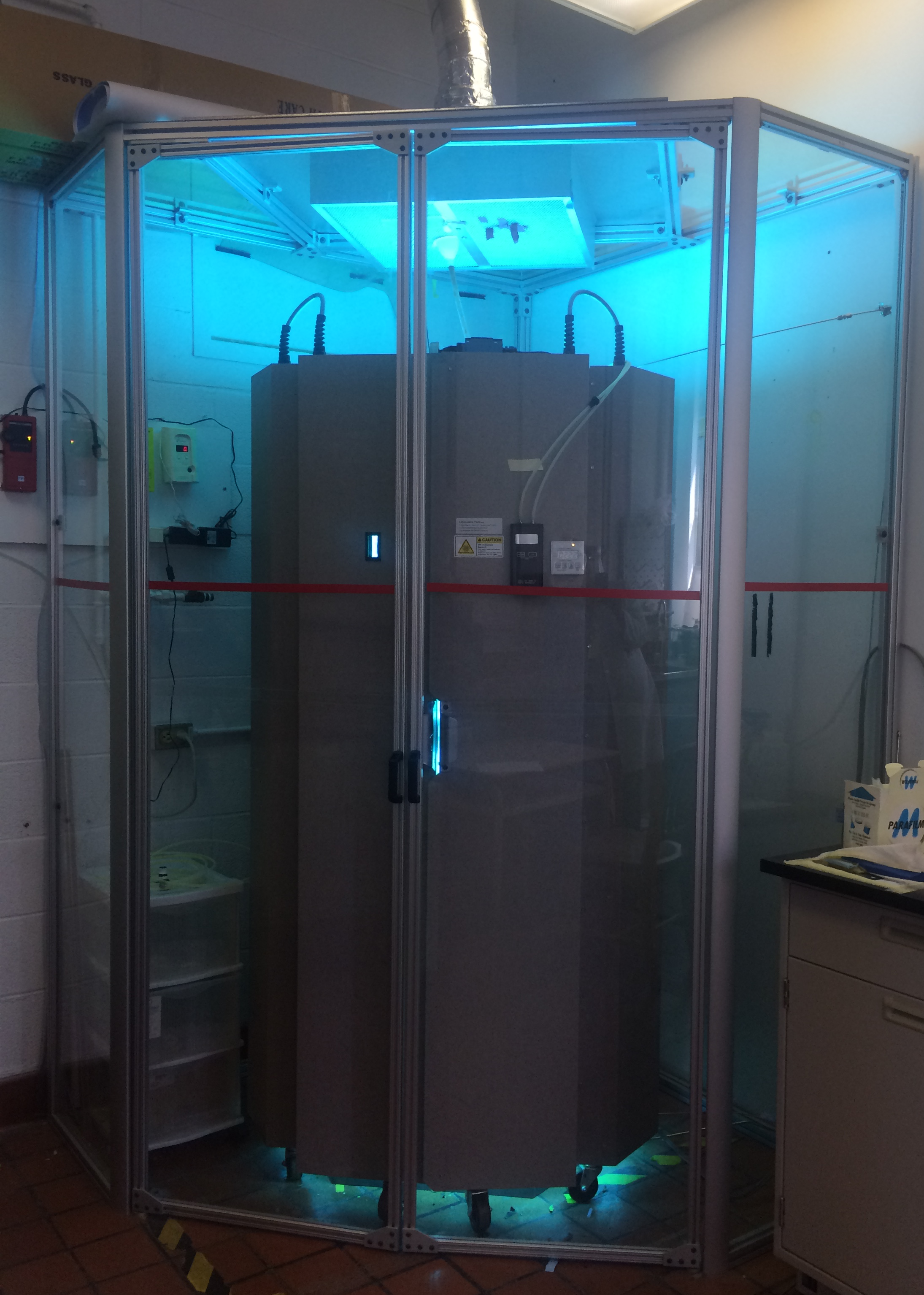 |
| --- |
| Figure 6S. Picture of the experimental set-up in operation: UV chamber with hexagonal cross section (67 cm=smallest dimension, 97 cm=largest dimension) and total cabinet height of 180 cm. |
